# Neuronal figure-ground responses in primate primary auditory cortex

**DOI:** 10.1016/j.celrep.2021.109242

**Published:** 2021-06-15

**Authors:** Felix Schneider, Fabien Balezeau, Claudia Distler, Yukiko Kikuchi, Jochem van Kempen, Alwin Gieselmann, Christopher I. Petkov, Alexander Thiele, Timothy D. Griffiths

**Affiliations:** 1 Biosciences Institute, Newcastle University Medical School, Newcastle upon Tyne, United Kingdom; 2 Cognitive Neuroscience Laboratory, German Primate Center, Göttingen, Germany; 3 General Zoology and Neurobiology, Ruhr University Bochum, Bochum, Germany

**Keywords:** auditory figure-ground segregation, scene analysis, stochastic figure-ground stimulus, auditory cortex, non-human primate, A1, rhesus macaque, auditory object, auditory figure, perceptual organization

## Abstract

Figure-ground segregation, the brain’s ability to group related features into stable perceptual entities, is crucial for auditory perception in noisy environments. The neuronal mechanisms for this process are poorly understood in the auditory system. Here, we report figure-ground modulation of multi-unit activity (MUA) in the primary and non-primary auditory cortex of rhesus macaques. Across both regions, MUA increases upon presentation of auditory figures, which consist of coherent chord sequences. We show increased activity even in the absence of any perceptual decision, suggesting that neural mechanisms for perceptual grouping are, to some extent, independent of behavioral demands. Furthermore, we demonstrate differences in figure encoding between more anterior and more posterior regions; perceptual saliency is represented in anterior cortical fields only. Our results suggest an encoding of auditory figures from the earliest cortical stages by a rate code.

## Highlights

• Neuronal figure-ground modulation in primary auditory cortex
• A rate code is used to signal the presence of auditory figures
• Anteriorly located recording sites encode perceptual saliency
• Figure-ground modulation is present without perceptual detection

## Graphical abstract

**Figure:**
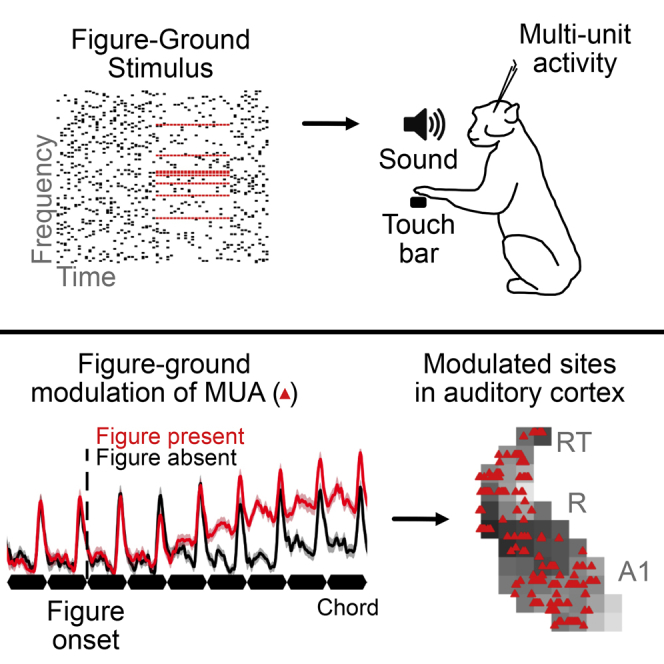

## Teaser

Using extracellular recordings of the auditory cortex, Schneider et al. demonstrate a neuronal correlate of complex auditory object segregation at the earliest stage, namely, the primary auditory cortex. A subset of recording sites encode the presence of coherent frequency elements with changes in their average activity.

## Introduction

Figure-ground segregation of natural scenes is essential for directing behavior, independent of the sensory modality. The perception of separated auditory objects in noisy scenes requires the brain to detect, segregate, and group sound elements that belong to the same figure or object (Bizley and Cohen, 2013; Griffiths and Warren, 2004). This process is related to stream segregation, for which cognitive processes cause perceptual organization of incoming sound. However, instead of hearing two distinct but equally relevant streams of sound, figure-ground segregation entails the emergence of foreground “figure” objects over background sounds referred to as “ground.” Temporal coherence between sound features has been proposed to drive this process (Shamma et al., 2011; Teki et al., 2013) by linking covarying features into a stable percept. Despite years of research, the neuronal processes that underly figure segregation are still mysterious.

The complexity of natural acoustic scenes can be modeled with stochastic figure-ground (SFG) stimuli, in which temporally coherent figure elements are segregated from random masker elements that overlap in frequency-time space. The detection probability of such synthetic figures by humans increases with the number of coherent frequency elements in the figure (O’Sullivan et al., 2015; Teki et al., 2011, 2013; Tóth et al., 2016). Moreover, figure detection correlates with speech-in-noise detection irrespective of hearing thresholds for pure tones (Holmes and Griffiths, 2019). Both SFG detection and speech-in-noise detection require cortical brain mechanisms (Holmes et al., 2019), highlighting the importance of central grouping mechanisms in normal hearing.

Human brain responses for figure-ground analysis have previously been investigated using EEG, MEG, and fMRI (O’Sullivan et al., 2015; Teki et al., 2011, 2016; Tóth et al., 2016). Neural ensemble activity in the auditory cortex, as measured with EEG and MEG, scales with figure coherence (O’Sullivan et al., 2015; Teki et al., 2016; Tóth et al., 2016). Changes in blood-oxygen-level-dependent (BOLD) fMRI activity occur in the non-primary auditory cortex (Schneider et al., 2018; Teki et al., 2011), although recent work also suggests the involvement of early auditory cortical areas (Holmes et al., 2019).

Neural correlates of human figure-ground segregation have been demonstrated even when attention is directed toward an irrelevant distractor task (Teki et al., 2011, 2016), consistent with pre-attentive processing. However, enhanced EEG responses occur during active listening (O’Sullivan et al., 2015), and differences in EEG activity between hit and miss trials support attentional effects (Tóth et al., 2016). In addition, figure-ground segregation seems to be susceptible to cognitive load across modalities; high visual load reduces auditory cortical activity to auditory figures (Molloy et al., 2018). Taken together, these findings suggest that the grouping of figure elements is possible without attention being directed to the sound but that the perception of the auditory object is facilitated by attentional modulation of brain responses.

The SFG paradigm allows the investigation of auditory figure-ground mechanisms in animal models as the stimulus is devoid of any species-specific meaning. Rhesus macaques (*Macaca mulatta*) are good models for human auditory scene analysis with homologous cortical organization (Baumann et al., 2013), comparable audiograms (Dylla et al., 2013; Jackson et al., 1999; Pfingst et al., 1975), equivalent pitch perception (Joly et al., 2014), tone-in-noise detection (Dylla et al., 2013), and comparable auditory streaming abilities (Christison-Lagay and Cohen, 2014; Selezneva et al., 2012). Crucially, figure-detection performance and cortical involvement during figure-ground segregation are comparable between macaques and humans (Schneider et al., 2018), suggesting highly similar sound processing capabilities.

Any neuronal mechanism for auditory figure-ground segregation needs to monitor the time-frequency space to perform the binding of foreground sound elements required for figure detection. Possible mechanisms for the representation of coherent figures might be based on single neurons or populations of neurons. Individual neurons can encode sound figures if their frequency selectivity covers all relevant features. A mechanism based on single neurons is unlikely in the primary auditory cortex, where the typical neuronal tuning (Recanzone et al., 2000) is not broad enough for the stimuli used in the present experiment. However, broadband responses are well established in the non-primary auditory cortex (Kikuchi et al., 2014; Rauschecker and Tian, 2004; Rauschecker et al., 1995), in which a mechanism based on single neurons is theoretically possible. Alternatively, coding of the emergence of figures from ground might be a property of groups of neurons in local circuits. In this scenario, the extent of the frequency selective region does not matter. Neurons would respond to sound elements within their receptive field but the combined responses of the population encode the figure.

In this experiment, we investigated figure-ground processing based on the extracellular activity of local groups of neurons in the auditory cortex. This reflects neuronal population mechanisms but with better spatiotemporal resolution than previous estimates of ensemble activity in rhesus macaques (Schneider et al., 2018). Based on the hemodynamic changes in the auditory cortex (Schneider et al., 2018), we hypothesized (1) the presence of neuronal modulation in response to auditory figures in anterolateral belt and parabelt regions, (2) the absence of such modulation in the primary auditory cortex (A1), and (3) the use of a rate code (i.e., changes in the rate of neuronal firing) that would explain the observed BOLD responses. The data presented here support a rate code. Surprisingly, we found highly distributed responses to auditory figures across the auditory cortex, including A1, thus establishing figure-ground representation at the earliest level of the auditory cortical hierarchy.

## Results

### Figure coherence is the decisive factor for perception

**Figure 1:**
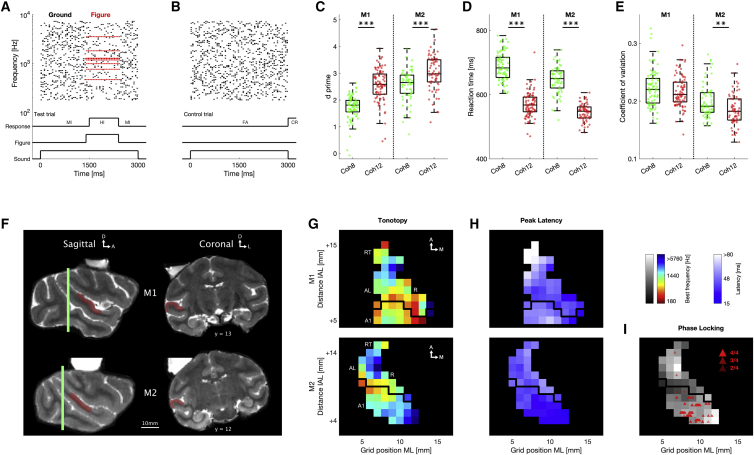
Summary of experimental paradigm, behavioral performance, and recording field (A) Schematic spectrogram of an example SFG stimulus. Figure elements are shown in red, and random ground elements in black. Line plots below indicate the 900-ms-long behavioral response window for the displayed stimulus as well as the behavioral outcome for touch bar release inside (HI, hit) and outside (MI, miss) of this time window. (B) Example control stimulus without figure. The trial was correctly performed, if no touch bar release occurred until sound presentation finished (CR, correct rejection). Otherwise, the trial was counted as false alarm (FA). (C–E) Behavioral detection performance of monkey 1 (M1; left, sessions: n = 87) and monkey 2 (M2; right, sessions: n = 67) for coherence levels of 8 (Coh8; green) and 12 (Coh12; red) elements, respectively. Only sessions with more than 200 trials were included. (C) Average d-prime values. (D) Mean reaction time. (E) Response variability measured by the coefficient of variation (standard deviation divided by mean). Stars indicate significance (two-sided Wilcoxon rank-sum test): ^∗∗^p < 0.01, ^∗∗∗^p < 0.001. (F) Structural T2 MRI of both subjects. Green vertical lines indicate location of interaural line. Distance of coronal sections from interaural line (mm) is indicated below slice. Recording chamber is filled with saline for visibility. Both recording chambers have a medial tilt (M1: 10 deg, M2: 15 deg) to allow easier access to the lateral auditory cortex. Auditory cortex is highlighted in red. A 10-mm scale bar is shown below. (G) Best frequency maps for M1 (top) and M2 (bottom). Color code indicates average best frequency across the surface of the superior temporal gyrus. Only sites with significant pure tone tuning are shown. Recording coordinates with unmodulated frequency response are not included. Y coordinates show distance to the interaural line (IAL). X coordinates show the grid position. Maps are smoothed with a 2 × 2-mm Gaussian kernel. The black line indicates the division boundary between anterior and posterior recording field based on low-frequency gradient reversal. Labels illustrate the estimated areal membership. (H) Latency map for M1 (top) and M2 (bottom). Color code illustrates average peak latency for each grid position. (I) Location of channels that exhibit significant LFP phase locking (red triangles) overlaid on M2's best frequency map. Strength of phase locking (no. of click train frequencies that elicit phase locking response) is indicated by transparency of triangle. See also Figures S1 and S2.

Two adult rhesus macaques (monkey 1 [M1]: male, 11 years; monkey 2 [M2]: female, 6 years) were trained to detect synthetic auditory figures in a noisy scene. Subjects listened to 3,000-ms-long SFG stimuli (60 chords, 50-ms duration) and used a touch bar to indicate in a go/no-go fashion whether they detected a target (Figures 1A and 1B). In 60% of trials, we presented a 1,000-ms-long (20 chords) figure with either 8 (Coh8) or 12 (Coh12) randomly chosen coherent frequency components—coherence levels that are highly salient to both humans (Teki et al., 2013) and macaques (Schneider et al., 2018). Figures were presented at a pseudorandom point between 300 ms and 2,000 ms after sound onset.

To investigate how auditory figures impact perception, we analyzed behavioral data of 154 sessions (M1: n = 87; M2: n = 67). D-prime, a discriminability index, was calculated to assess the detection performance for each session. Both subjects executed the figure detection task with high performance (Figures 1C–1E) in a similar manner to human behavior (Teki et al., 2013). For both monkeys, higher figure coherence increased d-prime values (Figure 1C; M1: standardized mean difference [SMD] = 1.23, two-sided Wilcoxon rank-sum test: Z = −8.10, p < 0.001; M2: SMD = 0.69, Z = −7.03, p < 0.001) and reduced reaction times (Figure 1D; M1: SMD = −1.65, two-sided Wilcoxon rank-sum test: Z = 8.10, p < 0.001; M2: SMD = −1.63, Z = 7.12, p < 0.001). Reaction times also differed on a trial-by-trial basis (linear mixed effects model: t_(36338)_ = −71.222, β = −0.024 ± 0.0003, p < 0.001), indicating that the number of coherent elements is a critical factor for the speed of object perception. The coefficient of variation was significantly different between coherence levels for M2 (Figure 1E; M1: SMD = −0.21, two-sided Wilcoxon rank-sum test: Z = 1.29, p = 0.1982; M2: SMD = −0.48, Z = 3.02, p < 0.01), for which a higher figure coherence caused a decrease in response variability. Taken together, these findings suggest that a greater number of coherent frequency components impacts the internal representation of auditory objects and causes enhanced, faster, and more reliable figure detection in a noisy scene. These data are in line with earlier behavioral observations (Schneider et al., 2018) and provide evidence for comparable figure-ground perception between humans and non-human primates.

### Recorded sites are mostly located in primary auditory (core) fields

**Figure 2:**
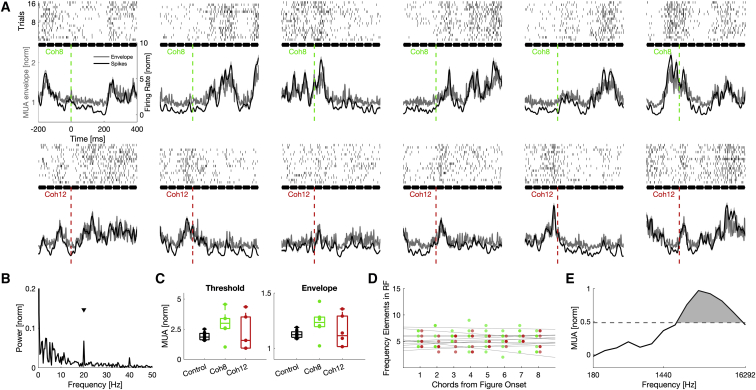
Figure-onset-aligned responses of an example site (A) Thresholded multi-unit spiking activity (black) and the multi-unit activity envelope (gray) in response to individual SFG stimuli are shown. Twelve different test stimuli were presented, of which 50% contained figures with Coh8 (green, top row). The remaining test stimuli contained figures with Coh12 (red, bottom row). Different plots correspond to individual figure-onset-aligned responses to different SFG stimuli. Figure onset is indicated by a dashed line at time zero. Raster plots are shown on top. Each row corresponds to a trial and each point within a trial to a single spike. Responses are baseline normalized, averaged over all trials. Single chords of the SFG stimulus are indicated in black. The standard error of the mean is illustrated as a shaded area. (B) Fast Fourier transform (FFT) of averaged MUA to SFG control stimuli for this recording site, which was normalized to the maximum power. The 20-Hz peak (black arrow) indicates rhythmic responses to each presented chord. (C) Mean MUA amplitude for stimuli of each coherence condition averaged in time window 201 to 400 ms after figure onset. Due to the small sample size (n = 6 for figure stimuli, n = 8 for control stimuli), no statistical test is shown. (D) Quantification of frequency elements that fall into the frequency-response area (see E) of the recording site for the first eight chords after figure onset shown for each stimulus. Color-coded points show stimulus-wise data. Gray lines demonstrate stimulus-wise linear regression of elements in RF. (E) Pure tone tuning curve of the example site averaged across sound intensities and then normalized to maximum response. Dashed line indicates half maximum. “Responsive” area indicated in gray.

Awake, head-restrained subjects performed the figure detection task, and neuronal spiking activity and local field potentials (LFPs) were recorded in the left auditory cortex of both monkeys (Figure 1F; Figure S1). Two to three (single or multi-contact) electrodes were used in most recording sessions. We assessed auditory cortical population activity, measured by the multi-unit activity (MUA) envelope signal in response to pure tones, click trains, and SFG stimuli. The envelope of the neuronal activity closely follows the dynamics of thresholded spiking (Figures 2A and 2C) but without losing subthreshold information of the acquired signal. This property is useful, as it reflects an unbiased estimate of pooled population activity from several single units in the vicinity of the electrode contact. The MUA envelope was calculated by rectifying the band-pass-filtered (0.6–9 kHz) neuronal signal before applying a lowpass filter with a 200-Hz cutoff.

To estimate the cortical location of each recording site, we assessed the best frequency (BF) and response latency to pure tones of various frequencies. Data from all recording coordinates were combined to create spatial maps of these functional response parameters (Figures 1G and 1H). In addition, structural MRI (Figure 1F) and either histology (M1, Figure S1) or phase-locked LFP responses to click trains (M2, Figures 1I and S2) were used to gauge the approximate electrode location. Low response latencies (Camalier et al., 2012) and phase locking to the click frequency (Lu et al., 2001; Oshurkova et al., 2008) were used as indicators of primary cortical areas. Although the applied methods allowed adequate distinction of cortical areas in an anteroposterior direction, we could not confidently separate the auditory core and belt regions. Thus, there is some degree of uncertainty regarding the areal membership of recorded sites. Based on the available evidence, we concluded that, in both subjects, most recording sites were located in the auditory core cortex (A1, rostral area [R], rostrotemporal area [RT]) and parts of the lateral belt, presumably the anterolateral area (AL) and rostrotemporal lateral area (RTL). We cannot explicitly exclude the contribution of other belt areas, e.g., the middle lateral area (ML) or the middle medial area (MM). M1’s chamber position (Figure 1F) and missing high frequency selectivity in the posterior recording region (Figure 1G) suggest that the recording field was slightly more anterior than that of M2.

### Figure-ground modulation in early auditory cortex

**Figure 3:**
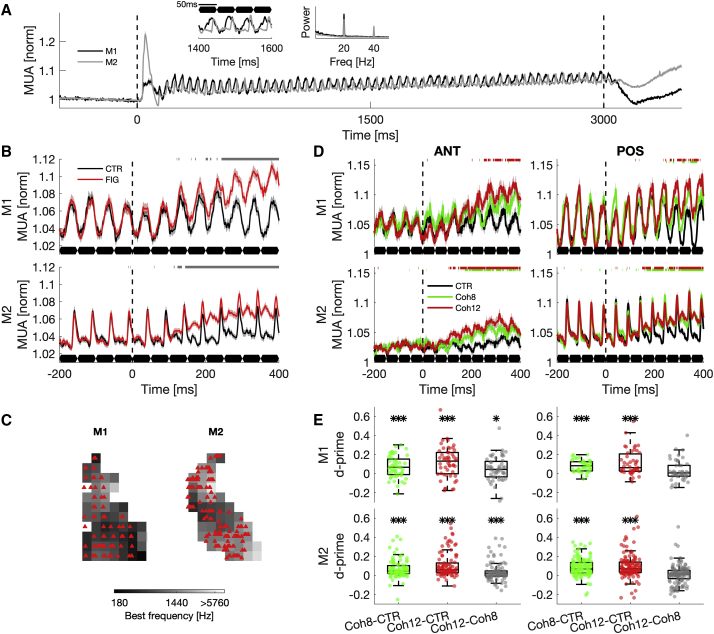
Average population responses of modulated sites to SFG stimuli (A) Average MUA in response to control stimuli for both M1 (black) and M2 (gray). Inset shows zoomed response to four chords (relative to sound onset) and the FFT of the average response, which was normalized to the maximum power. (B) Figure-onset-aligned population time course of modulated recording sites for M1 (top) and M2 (bottom) to auditory figures (red) and control condition with no coherent elements (black). Shaded regions represent the standard error of the mean. Figure onset is indicated as a dashed line. SFG chords are outlined in black below. Significantly different responses for figure versus control conditions are depicted in gray above (two-sided Wilcoxon rank-sum test, p < 0.05, FDR corrected). (C) Spatial maps of the recording field indicate the location of individual figure-responsive sites (red triangles). In contrast to the underlying map, these coordinates are corrected for recording angle and depth to better visualize the spread of modulated recording sites. This can result in locations outside the visible map. (D) Figure-onset aligned population time course of modulated sites for anterior (ANT, left) and posterior (POS, right) recording field for each subject (M1: top, M2: below). Average MUA to Coh12 (red), Coh8 (green), and no coherent elements (CTR, black) is shown. Similar conventions as (B). (E) Color-coded boxplots show neuronal d-prime for each subject (M1: top, M2: below), based on averaged, baseline-normalized MUA between chords 5 to 8 (201 to 400 ms) after figure onset. p values are FDR corrected. Stars indicate significance (two-sided Wilcoxon rank-sum test): ^∗^p < 0.05, ^∗∗^p < 0.01, ^∗∗∗^p < 0.001. See also Figure S3.

Based on previous work (Schneider et al., 2018; Teki et al., 2011), we hypothesized that neuronal correlates of figure-ground modulation would emerge in higher cortical centers, e.g., the anterolateral belt and parabelt. To test this hypothesis, we presented a new set of randomly drawn SFG stimuli in each recording session (total number of sessions = 125, M1: n = 66, M2: n = 59). Stimuli contained 60 chords with a duration of 50 ms that were presented at a rate of 20 Hz with no gap in between. This broadband signal consistently drove MUA, evidenced by a strong 20-Hz oscillation of the neuronal signal (Figures 2B and 3A).

For each recorded site, we analyzed two distinct time windows as following: “onset”-aligned data refer to the period 201 ms to 400 ms after figure onset, and the “response”-aligned time window describes cortical activity −300 ms to −100 ms prior to touch bar release. Depending on the reaction time of the respective trial, these time windows might overlap slightly. In figure trials without touch bar release (miss), response-aligned data were referenced to the last 200 ms of the figure presentation period. Neuronal activity in control trials, in which no figure was present, was aligned to a pseudorandomly chosen 200-ms-long period.

To identify brain activity that was modulated by auditory figures, we used a two-sample t test to compare response-aligned MUA in correctly performed figure and control trials. A subset of recorded sites showed significantly modulated MUA in response to auditory figures (figure versus control, two-sample t test, p < 0.01; site count, M1: n = 99, 29.6%; M2: n = 228, 36.7%). Unless otherwise stated, we subsequently analyzed these modulated multi-units.

We first focused on MUA after the onset of a figure (Figure 2). Generally, cortical responses to different stimuli were highly variable (Figures 2A and 2C). However, on average, the figure-onset-aligned population signals of responsive recording sites revealed an evolving increase in MUA in both subjects (Figure 3B). A comparison between figure and control conditions revealed significantly increased MUA shortly after the onset of the coherent figure elements (M1: SMD = 0.32, two-sided Wilcoxon rank-sum test: Z = 6.50, p < 0.001; M2: SMD = 0.23, Z = 11.32, p < 0.001), suggesting the temporal coherence of frequency elements as the likely cause. In contrast to expectations from hemodynamic responses (Schneider et al., 2018; Teki et al., 2011), we demonstrate that figure-modulated MUA was widely distributed across the recording field (Figure 3C), including the primary auditory cortex (A1). Modulated sites show no spatial clustering toward the anterolateral part of the auditory cortex. Thus, recording sites at the earliest cortical stage represent temporally coherent frequency elements in a noisy scene.

### Response differences between anterior and posterior recording field

Previous fMRI studies (Schneider et al., 2018; Teki et al., 2011) have highlighted the contribution of the anterolateral, non-primary auditory cortex during figure-ground segregation. Therefore, we investigated whether neuronal figure-ground modulation would be more pronounced in anteriorly located recording sites. We quantified neuronal population responses in different parts of the auditory cortex by using the tonotopic low-frequency gradient reversal to subdivide the recording field into an anterior (site count, M1: n = 52; M2: n = 98) and a posterior section (site count, M1: n = 47; M2: n = 130). As described above, the posterior recording fields mainly covered A1 and did not include areas of the caudal belt. The anteriorly located recording sites encompassed area R and to some extend RT, RTL, and AL. As mentioned, we can only approximate areal membership based on the location of recording sites relative to the low-frequency gradient reversal.

We calculated neurometric d-prime, which measures activity differences between stimulus conditions divided by the standard deviation across stimulus conditions, for each modulated recording site to make inferences about the magnitude of figure-ground modulation between different recording sites. Based on the baseline-normalized, onset-aligned MUA under figure and control conditions, we did not find a significant difference in neurometric d-prime between anterior and posterior recording fields (M1: SMD = −0.02, two-sided Wilcoxon rank-sum test: Z = 0.05, p = 0.9614; M2: SMD = −0.01, Z = −1.52, p = 0.1283), suggesting overall similar neuronal modulation magnitudes (Figures 3D and 3E).

Previous human imaging studies have demonstrated that figure coherence parametrically changes brain activity (O’Sullivan et al., 2015; Teki et al., 2011, 2016). To assess the neuronal encoding of figure coherence, we further quantified coherence-driven figure-ground modulation for each modulated site by calculating neurometric d-prime values between the two tested figure conditions (Coh8 versus Coh12). We show that multi-unit responses between the tested coherence levels were different between recording fields (Figure 3E, two-sample t test, p < 0.05). Only in anterior regions were neurometric d-prime values different from zero (M1, Anterior recording region (ANT): median = 0.044, two-sided Wilcoxon rank-sum test: Z = 2.77, p < 0.05; Posterior recording region (POS): median = 0.008, Z = 1.15, p = 1; M2, ANT: median = 0.020, Z = 4.00, p < 0.001, POS: median = 0.014, Z = 1.70, p = 1, FDR-corrected), suggesting coherence-dependent MUA modulation in the anterior core/belt areas, with larger response modulation for higher coherence levels. The observed effects were not due to a quantitative change in frequency content in the receptive field of the sites (example site: Figures 2D and 2E, population: Figure S3A). MUA modulation, despite steady receptive field stimulation, indicates that the temporal coherence of figure elements, corresponding to increased regularity of the stimulus, drove this cortical response. Modulation latencies were similar across coherence levels and cortical subfields (Figure S3B, coherence: SMD = 0.07, two-sided Wilcoxon rank-sum test: Z = 0.60, p = 0.5465; field: SMD = 0.06, Z = 0.78, p = 0.4334), with median modulation latencies of two to three chords (Coh8: 112 ms, Coh12: 108 ms), suggesting no temporal processing differences between populations in different subfields.

### Stable modulation of cortical activity

**Figure 4:**
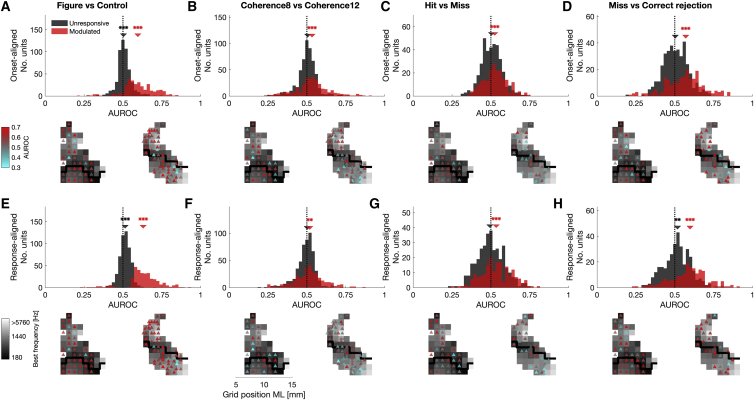
Summary of cortical response modulation Data of both subjects were pooled. Averages of onset-aligned (a-d, 201 to 400ms past figure onset) and response-aligned MUA (e-h, -300 to -100ms prior to touch bar release) were used for calculation of area under receiver operating characteristic (AUROC). Histograms show distributions of modulated (p < 0.01, red) and unresponsive recording sites (black). Below: Modulated units overlaid on tonotopic map. Color coding corresponds to effect size. (A and E) Figure-ground modulation. (B and F) Modulation based on figure coherence. (C and G) Modulation based on behavioral detection of temporally coherent elements. Only recordings with at least 20 miss trials were included. (D and H) Modulation based on temporal coherence without detection. Arrows indicate median of distribution. Data were tested against 0.5 with a two-sided Wilcoxon rank-sum test. All p values are FDR corrected. Color-coded stars indicate significance: ^∗^p < 0.05, ^∗∗^p < 0.01, ^∗∗∗^p < 0.001. See also Figures S4 and S5.

Sustained stimulation usually results in adaptation of cortical responses (Recanzone, 2000; Ulanovsky et al., 2004). However, compared to random sequences, auditory regularity has been shown to increase brain activity (Barascud et al., 2016; Sohoglu and Chait, 2016; Southwell et al., 2017). We were interested whether auditory figures would produce adaptation or stable modulation of neuronal activity across the auditory cortex to signal the presence of an object. To this end, we calculated the area under the receiver operating characteristics (AUROCs) for onset- and response-aligned MUA of each recorded site. AUROC values of 0.5 indicate indistinguishable distributions, whereas a value of 0 or 1 corresponds to perfectly separated distributions of neuronal responses. Data of both animals were pooled. This analysis confirms significant figure-ground modulation in both time windows, suggesting a stable modulation of cortical activity. The distribution averages were significantly shifted toward values larger than 0.5 (Figures 4A and 4E; modulated sites: onset-aligned: median = 0.5961, two-sided Wilcoxon rank-sum test: Z = 13.08, p < 0.001; response-aligned: median = 0.6302, Z = 13.91, p < 0.001; unresponsive sites: onset-aligned: median = 0.5036, Z = 3.60, p < 0.001; response-aligned: median = 0.5144, Z = 8.83, p < 0.001, FDR corrected), suggesting that the majority of modulated sites responded with excitation to auditory figures (onset-aligned: M1: 84.7%, M2: 93.4%; response-aligned: M1: 88.8%, M2: 94.7%). Even though most recording sites showed enhanced responses, we also demonstrate reduced MUA in response to auditory figures (Figures 4A and 4E). The fact that the distribution of unresponsive sites differed from 0.5 can be explained by the conservative inclusion threshold (two-sample t test, p < 0.01).

Response-aligned AUROC values were significantly higher than onset-aligned data (two-sided Wilcoxon rank-sum test: Z = 5.40, p < 0.001). The SMD between response-aligned and onset-aligned data is positive (SMD = 0.3228), indicating that AUROC values were higher shortly before subjects release the touch bar. This increase in AUROC values suggests a continuous ramp-up in MUA after the onset of an auditory figure.

Significant differences in response to stimuli with different figure coherence levels were observed for both onset- and response-aligned data when pooled across monkeys (Figures 4B and 4F; modulated sites: onset-aligned: median = 0.5332, two-sided Wilcoxon rank-sum test: Z = 5.95, p < 0.001; response-aligned: median = 0.5184, Z = 2.92, p < 0.01; unresponsive sites: onset-aligned: median = 0.5051, Z = 1.50, p = 1; response-aligned: median = 0.5022, Z = −1.34, p = 1, FDR corrected), suggesting a consistent encoding of perceptual saliency in figure-responsive cortical MUA.

Broad neuronal frequency tuning could be the reason for higher responses to figures with more coherent elements. However, we did not find a consistent relationship between figure-ground modulation and the width of the multi-unit tuning curve (Figure S3C), suggesting that broadband tuning does not facilitate figure processing. Note that multi-unit frequency selectivity might not reveal such effects.

### Figure-ground modulation without perceptual detection

Previous work has demonstrated differences in brain responses in passive versus active listening conditions and between behavioral categories (O’Sullivan et al., 2015; Tóth et al., 2016). We wondered whether we could observe similar response differences between the types of behavioral trial outcomes.

Neuronal responses were compared between miss and correct rejection trials to investigate if elevated spiking responses occur even without behavioral detection. To this end, we calculated the AUROC based on MUA of the two trial outcomes (miss and correct rejections). Only recording sites with more than 20 miss trials were included in the analysis. MUA of figure-responsive sites differed significantly between trial types, with higher AUROC in miss trials (Figure 4D; Figure S4; modulated sites: onset-aligned: median = 0.5705, two-sided Wilcoxon rank-sum test: Z = 6.68, p < 0.001; response-aligned: median = 0.5982, Z = 8.26, p < 0.001; unresponsive sites: onset-aligned: median = 0.5033, Z = 1.16, p = 1; response-aligned: median = 0.5177, Z = 3.28, p < 0.01; FDR corrected). Elevated cortical responses in miss trials suggest that even without behavioral detection, auditory cortical neurons responded to temporally coherent elements.

When the monkeys detected a figure (“hit”), MUA of figure-responsive sites was significantly increased compared to trials in which animals did not report the target (miss; Figure 4C; onset-aligned: median = 0.5247, two-sided Wilcoxon rank-sum test: Z = 4.78, p < 0.001; response-aligned: median = 0.5342, Z = 4.50, p < 0.001; unresponsive sites: onset-aligned: median = 0.5038, Z = 0.96, p = 1; response-aligned: median = 0.4936, Z = −0.63, p = 1; FDR corrected), suggesting a link between spiking activity of figure-responsive sites and object perception. However, this effect was mainly driven by one subject (two-sided Wilcoxon rank-sum test, onset-aligned: M1: median = 0.5442, Z = 5.69, p < 0.001, M2: median = 0.5113, Z = 1.00, p = 0.3181; response-aligned: M1: median = 0.5458, Z = 4.63, p < 0.001, M2: median = 0.5131, Z = 1.69, p = 0.0915). Therefore, our findings are not conclusive given the contradicting data of the two monkeys.

Neuronal activity on false alarms trials could not be investigated, as there were consistently very few trials that prevented analysis.

### Figure-ground modulation not based on motor-related activity

An inherent flaw of the go/no-go task design is the behavioral response difference between stimulus conditions. That is, whenever the monkey detected a figure, it had to release a touch bar. In contrast, subjects were supposed to maintain contact with the touch bar during control trials in which no figure could be detected. Thus, in addition to the difference in auditory stimulation, these conditions differed with respect to arm movements.

To control for the contribution of motor-related signals in the auditory cortex, we conducted 10 control experiments with 1 subject (M2). In these additional recording sessions, the monkey was rewarded for self-paced touch bar releases in complete silence. We looked at the activity difference in the neuronal activity between a response-aligned time window (-301 to -100 ms prior to touch bar release) and a baseline time window (-501 to -300 ms prior to touch bar release). We did find consistently elevated neuronal activity shortly before the response (Figure S5; SMD = 1.0769, two-sided Wilcoxon rank-sum test: Z = 4.10, p < 0.001), suggesting a contribution of motor-related activity in the analyzed data. However, judging by the difference of the average activity in the respective time window, the magnitude of this effect was rather small (mean MUA difference ± SEM: 0.0189 ± 0.0038) compared to the difference between figure and control (Figure 3B).

Despite elevated neuronal activity prior to touch bar release, the figure-ground modulation reported here is unlikely to result from motor activity due to several reasons. First, we have shown that neuronal activity, on average, ramps up about 200 ms after figure onset (Figure 3B), which is much earlier than the average reaction time of the subjects (Figure 1D). Second, trials in which a figure was presented but subjects failed to respond (miss) showed significantly higher MUA activity than the control condition (no figure, no motor response, see Figure 4). Third, the majority of recorded sites was not modulated by auditory figures. If the observed figure-ground modulation would be caused by motor-related activity, all recording sites would show different figure versus control responses. Lastly, but most importantly, the increased MUA response seems to be caused by a motor or grounding artifact that can be observed at the point of touch bar release. In some trials, this artifact occurred slightly before the logged touch bar release timestamp and might have extended into the analysis window (Figure S5B), suggesting that the monkey already moved the hand considerably without breaking contact with the touch bar. When we averaged the neuronal data across all trials, this artifact seems to cause the observed ramping in the neuronal response. However, without precise tracking of hand/arm movements, we cannot make a definitive claim about the origin of the elevated neuronal activity.

## Discussion

We investigated the neuronal correlates of figure-ground segregation by recording extracellular MUA of auditory cortical neurons. Auditory figures produced robust changes in neuronal firing for a subpopulation of recording sites across the auditory cortex, showing no sign of spatial clustering (Figures 3B and 3C). The modulation of cortical activity was not driven by the frequency content of the stimulus (Figure S3A), suggesting that clusters of neurons across the cortical hierarchy signaled the presence of a figure with alterations (generally increases) in their average activity. Thus, our findings suggest that modulated sites signaled auditory figures with a rate code. The simultaneous modulation of neuronal firing across cortical locations is consistent with the temporal coherence model (Shamma et al., 2011). Whether the changes in neuronal responses we observed are due to single neuron modulation, a local circuit, or a distributed population code cannot be answered by our experiment and needs further investigation.

Our data provide evidence that primary cortical neurons do already respond to conjunctive sound features. A1 sites detected temporally coherent frequency elements that had no simple mathematical relationship to each other (Figures 3C–3E, 4A, and 4E), unlike previous work that identified A1 responses to harmonically related elements (Feng and Wang, 2017). These results accord with recent findings showing that perceptual organization depends on the primary auditory cortex for the segregation of repeated noise stimuli (Saderi et al., 2020) and synchronous tone sequences (Lu et al., 2017). In addition, dynamic causal modeling has demonstrated disinhibition of the primary core during the presentation of auditory figures (Holmes et al., 2019).

Previous fMRI studies that directly investigated the contrast of figure versus control stimuli did not describe a significant change in BOLD responses in the primary auditory cortex (Schneider et al., 2018; Teki et al., 2011). However, Schneider et al., (2018) tested naive monkeys. Earlier studies have shown modulation of A1 neurons during task engagement (i.e., active listening), which was absent during passive listening (Atiani et al., 2009, 2014; Bagur et al., 2018; Fritz et al., 2003; Lu et al., 2017). Thus, auditory core responses to the complex grouping of random elements at the earliest cortical stage may be a result of active task engagement. Alternatively, BOLD responses might have been too insensitive to pick up figure-ground responses in core areas, as only approximately 30% of recorded cell clusters showed MUA modulation. With the current detection paradigm, we demonstrate MUA modulation in trials without behavioral detection of the figure (Figure 4D, miss versus correct rejection trials) that provides further evidence for pre-attentive figure segregation (Schneider et al., 2018; Teki et al., 2011). Taken together, our findings support an involvement of the primary auditory cortex in complex figure-ground segregation.

Our work confirms that the number of coherent figure elements has an impact on the auditory cortical response (Figure 3E), as has been shown in earlier studies (O’Sullivan et al., 2015; Teki et al., 2016; Tóth et al., 2016). However, sites in the posterior part of the recording field showed no coherence-dependent response modulation. In contrast, cortical population activity in the anterior auditory cortex shows a strong link between the number of coherent figure elements and cortical firing. Thus, anterior auditory regions seem to code for the perceptual salience of figures, whereas posterior fields might only signal the presence of a figure. One interpretation of these results is a hierarchical encoding of object features, for which anterior regions represent a larger range of figure properties. A stepwise, rostrally directed flow of information has been proposed for the highly interconnected core areas (Kaas and Hackett, 2000; Scott et al., 2017a). Other findings suggest a hierarchical relationship between anterior and posterior auditory cortex (Kikuchi et al., 2010) in which increased complexity of sensory representation occurs in anterior areas that have been linked to object recognition (Jasmin et al., 2019). However, highly interconnected auditory cortical areas (Hackett et al., 2014; Scott et al., 2017a), as well as differential thalamic projections (Scott et al., 2017b), might contradict a simple, linear processing strategy.

Previous studies based on classical tone-sequence streaming paradigms led to the argument that stream segregation depends on tonotopically organized narrowband responses in the primary auditory cortex (Fishman et al., 2001, 2004, 2017). This argument was based on neuronal responses to tones located on either the slopes or the peak of the tuning curve. Alternating tone sequences with a larger frequency difference between tones showed more effective tonotopic separation, although a similar trend could also be observed for synchronous stimuli (Fishman et al., 2017). Our data suggest that the A1 population can indeed segregate synchronous target frequencies from random, irrelevant information. However, the stimulus containing the figure and the control stimulus without the figure likely activate the same populations of frequency-tuned neurons, making a theory of segregation based on the activation of distinct populations hard to sustain.

Although the population separation model seems insufficient to explain figure-ground processing, it becomes clear that A1 is indeed involved in cognitive processes. For instance, experiments using alternating tone sequences in awake, behaving monkeys have demonstrated that A1 activity correlates with the choice of the subject (Christison-Lagay and Cohen, 2018). In addition, a series of experiments, in which a light flash promotes either stream integration or separation, has shown that auditory cortical activity of attentive rhesus monkeys is related to auditory streaming (Selezneva et al., 2018). Auditory cortical neurons also modulate their activity based on the frequency differences in coherent two-tone sequences (Elhilali et al., 2009; Fishman et al., 2017). Combined with our findings, we conclude that A1 carries information that might be relevant to perceptual organization.

Previous work (Teki et al., 2013) considered whether figure detection might be based on temporal coherence detection or adaptation of tonotopic responses in frequency channels and provided behavioral evidence for the former. In contrast, other studies have suggested that changes in the temporal correlation of a stimulus causes neuronal adaptation by gain control mechanisms (Natan et al., 2016) by which neuronal firing adapts to the statistical context to efficiently encode different sound environments. In our experiment, the increases in MUA reflecting coherence would not support a mechanism based on neuronal adaptation or gain control.

Cortical responses to figure-ground stimuli (Teki et al., 2016) and synchronous (Lu et al., 2017) and regular tone sequences (Barascud et al., 2016) require a build-up period. Here, we show that, on average, it takes about two chords (100 ms) for most recording sites to detect the changes in stimulus statistics, which is in the same range as previously reported MEG and EEG data (O’Sullivan et al., 2015; Teki et al., 2016). However, modulation latencies can be longer than 400 ms (eight chords). This could reflect a cortical circuit, feedback connections, or simply a very small figure-ground effect that makes modulation latency estimation imprecise. No latency differences between coherence levels were found in our study, suggesting equal timescales of cortical processing independent of the magnitude of change in stimulus statistics.

In summary, our results indicate that a subset of recording sites in the auditory cortex signal the presence of a figure in a scene. The distribution of the observed response modulation challenges previous models suggesting specialization for figure-ground analysis that emerges only in the high-level auditory cortex. Our data demonstrate figure-ground modulation at the earliest cortical stage.

## STAR★Methods

### Key resources table

**Table:** 

REAGENT or RESOURCE	SOURCE	IDENTIFIER
**Experimental models: organisms/strains**		

Rhesus macaques	Centre for Macaques (CFM), Porton Down, Salisbury, Wiltshire	Macaca mulatta

**Software and algorithms**		

MATLAB	https://www.mathworks.com/	2020b
Amide	http://amide.sourceforge.net	1.0.5
Figure scripts	https://github.com/FelixSchneider1990/FigureGround_2021_CellReports	N/A
Analysis scripts	https://github.com/FelixSchneider1990/FigureGround_Ephys_Analysis	N/A

**Deposited data**		

Preprocessed MUA data	https://osf.io/5QJCK	DOI 10.17605/OSF.IO/5QJCK

### Resource availability

#### Lead contact

Further information and requests for resources should be directed to and will be fulfilled by the lead contact, Felix Schneider (fschneider@dpz.eu).

#### Materials availability

This study did not generate new unique reagents.

#### Data and code availability

Original data generated in this study have been deposited to Open Science Framework (DOI 10.17605/OSF.IO/5QJCK, https://osf.io/5qjck/).

The MATLAB code generated during this study is available at GitHub (https://github.com/FelixSchneider1990/FigureGround_2021_CellReports and https://github.com/FelixSchneider1990/FigureGround_Ephys_Analysis).

### Experimental model and subject details

#### Animals

Two adult rhesus macaques, Monkey 1 (‘M1’, Male, 11yrs, 11kg) and Monkey 2 (‘M2′, Female, 6yrs, 7kg), participated in this study. Animals were group housed. The operant training procedure is described elsewhere (Schneider et al., 2018), where both monkeys contributed behavioral data. Data reported here were recorded between 6 and 24 months after learning the task.

A circular PEEK chamber (17mm ID) was implanted over the left hemisphere with a 10 degree (Monkey 1) or 15 degree (Monkey 2) medial tilt to allow access to the left auditory cortical areas. Structural and functional MRI scans were used to position the chamber. The chamber implantation procedure is described elsewhere (Thiele et al., 2006). During testing periods, animals were kept under fluid-controlled conditions. Fluid control was within ranges which do not negatively affect animal’s physiological or psychological welfare (Gray et al., 2016).

All procedures performed in this study were approved by the UK Home Office (Project License: 70/7976) and by the Animal Welfare and Ethical Review Body at Newcastle University. All experiments comply with the UK Animals Scientific Procedures Act (1986) on the care and use of animals in research, with the European Communities Council Directive on the protection of animals used in research (2010/63/EC).

### Method details

#### Figure detection task

The behavioral task in this study is nearly identical to earlier experiments (Schneider et al., 2018). Monkeys were seated in a primate chair (Christ Instruments) in a sound-attenuated chamber with a touch bar and a gray screen (Acer K242HL) in front of them. Trials were initiated by bar touch. After a 500ms baseline period, a stochastic figure-ground (SFG) stimulus was presented. We used two speakers (Creative GigaWorks T20 Series II) that were placed at a 45-degree angle with respect to the midline of the animal at a distance of about 80cm. The animals were trained to signal the presence of a figure by touch bar release. Independent of the behavioral outcome of the trial, sounds were kept on for the entire stimulus duration (3 s). Visual feedback was given immediately after response. The color of the screen changed either to green for correctly performed trials or to red for error trials. In hit trials, the amount of reward given was reaction time dependent. Faster responses led to higher volumes of juice. For correct rejections, the amount of reward was fixed. Reward was always administered after the stimulus presentation period. For error trials (Miss and False alarm), a time-out of two seconds was imposed in addition to the inter-trial interval (2 s). If three error trials occurred in a row, a longer time-out of ten seconds was imposed.

In each recording session, 20 randomly selected stimuli, of which 60% contained a figure, were presented in pseudo-random order to ensure an equal number of presentations. We presented signals with two coherence levels (figures composed of 8 & 12 frequencies, equal probability).

#### Control experiment: Bar release task

Ten additional recording sessions were performed to assess the contribution of motor-related signal in the auditory cortex. Monkey M2 was rewarded for self-paced touch bar releases in complete silence. Responses were rewarded with a fixed amount of juice as long as the inter-response interval exceeded 1000ms. Otherwise, no reward was given. We used three single contact tungsten electrodes (FHC, Bowdoin, ME) per session, with recording sites scattered across the recording field. In addition, 200ms-long white noise bursts were presented to test whether recorded sites were driven by sound. All recording sites were sound-responsive. No additional training was required for this task. Control experiments were performed after the data acquisition of the main experiment was completed.

#### Acoustic stimuli

##### Stochastic figure-ground stimuli

Similar to previous behavioral work (Schneider et al., 2018), SFG stimuli were created at a sampling rate of 44.1 kHz with MATLAB (The Mathworks Inc., Natick, MA) and consisted of a sequence of 50ms long chords that were defined as the sum of multiple randomly selected pure tone elements. Frequencies were drawn from a pool of 129 evenly spaced frequencies (1/24 octave between successive frequencies) on a logarithmic scale between 179 Hz and 7246 Hz. The onset and offset of each chord were shaped by a 10ms raised-cosine ramp with no gap in between chords. 60% of stimuli included a sequence of repeated elements in a specified number of randomly selected frequency channels (‘Figure’). The remaining signals comprised randomly shuffled elements only (‘Control’).

SFG stimuli contained 60 chords (3 s in duration) and had a fixed number of elements per chord (n = 15). In contrast to earlier studies (Teki et al., 2011, 2013, 2016), extra elements (coherent versus shuffled) were not added on top but incorporated into the existing stream of chords. This way, any sound level cues at the onset of the figure were eliminated and consistent broadband power across chords was ensured. Stimuli were presented at 65 db SPL but due to the non-linearity of the speakers, sound intensity was variable (±3dB SPL). Figure onset times were randomized between 0.3 and 2 s after trial start. The number of stimulus repetitions varied based on the overall number of performed trials.

##### Pure tones

A total of 14 pure tones (200ms long, half-octave step-width [180Hz – 16292Hz]) were presented during every recording session. A 10ms cosine on- and off-ramp was applied to all signals. Tones were presented at three different intensities (50dB, 60dB, and 70dB SPL). A minimum of 10 repetitions per stimulus condition was obtained in each session.

##### Click trains

Monophasic, 200ms long click trains with varying frequencies (25 Hz, 50 Hz, 75 Hz and 100Hz) were presented at 80dB SPL. Each pulse had a duration of 2ms. A minimum of 10 repetitions per condition was obtained for a number of recordings with Monkey 2 (36/101 recordings, 89% of recorded channels).

Pure tones and click train stimuli were presented in an alternating block design. Per block, each stimulus was presented once. Within each block, the presentation order was randomized.

##### White noise

200ms long white noise stimulation was created by generating a random number vector. This vector was produced online and presented to the animal at 80dB SPL. A minimum of 30 repetitions was obtained in each session of the touch bar release experiment.

#### Neurophysiological recordings

Multi-unit activity and local field potentials were recorded by advancing one to three microelectrodes (0.2-5MΩ) into the auditory cortex by means of a remotely (CMS Drive, NAN Instruments) or manually controlled microdrive (MO97 Oil Hydraulic Micromanipulator, Narishige). Epoxylite-coated tungsten electrodes (FHC, Bowdoin, ME), custom-built glass-coated tungsten electrodes or 16-channel electrode arrays (V-probe, Plexon, Dallas, TX) were used for recordings. Stainless steel guide tubes (23 ga or 26 ga) were used to penetrate the dura mater. Custom-made PEEK grids (1x1mm or 0.8x0.8mm) were oriented approximately parallel to the anteroposterior axis and served as spatial reference for the electrode position. The signal was referenced to the guide tube or electrode shaft (V-probe), amplified, filtered (LFP: 1-300Hz, Spiking: 600-9000Hz), digitized (LFP: 1kHz, Spiking: 32kHz) and recorded via a 32-channel Digital Lynx SX acquisition system (Neuralynx, Cheetah 5.6 software). Anatomical landmarks (lateral sulcus), noise bursts and natural sounds were used to identify that the auditory cortex was reached.

Stimulus presentation, behavioral control and reward administration was controlled with an in-house program written in Python 2.7, which is partly based on Psychopy (Peirce, 2007) on Ubuntu 16.04 LTS via a DAQ-LabJack U6-Pro Interface. Recording sessions started with the figure detection task. The battery of sounds used to assess the tuning of the recording site was presented after the subject stopped working. A microphone (Audio Technica U841R with AT8531 power module) placed in front of one of the speakers (Creative GigaWorks T20 Series II) recorded the sound environment within the sound-attenuated booth. This signal was used to correct the sound onset timestamps offline for every trial by adding the delay period between timestamps and physical sound onset.

### Quantification and statistical analysis

#### Behavior

Behavioral performance of 154 sessions (M1: n = 87; M2: n = 67) was evaluated via d-prime. This sensitivity index provides a measure of separation between signal and noise distribution and takes all possible behavioral responses into account (Stanislaw and Todorov, 1999). D-prime was calculated in the following way: d’ = Z(Hit rate) - Z(False alarm rate), where Z is the inverse of the standard normal cumulative distribution function of hit rate and false alarm rates, respectively. To assess the effect of figure coherence on reaction times (Period between figure onset and touch bar release) on a trial-by-trial basis, a linear mixed effects model was constructed with figure coherence defined as a fixed effect. Random intercepts were included for each subject and session to account for repeated-measurements. This model was tested against an intercept-only model without coherence as a factor by means of maximum likelihood ratio tests. In addition, reaction times were analyzed by comparing the coefficient of variation between coherence levels. The coefficient of variation, a measure of data dispersion, is the ratio of standard deviation divided by the mean. D-prime values, mean reaction times, and response variability was tested across all included recording sessions with a Wilcoxon signed rank text for each subject individually.

#### Neuronal activity

We analyzed neuronal activity of 125 recording session (Number of sessions M1: n = 66, M2 n = 59). The envelope of the multi-unit activity was calculated by taking the absolute values of the band-pass filtered analog signal (600-9000Hz). The signal was then down-sampled to 1kHz after low-pass filtering using a third-order Butterworth filter with a 200Hz cut-off frequency. Spike density functions were computed by fitting a Gaussian curve with a width of 5ms to each detected spike. Subsequently, spike-wise Gauss curves were summed and averaged over stimulus repetitions. Data were baseline-normalized using the 400ms window prior to sound onset for further analysis.

To create spatial maps of the recording field, neural responses to pure tone stimuli were evaluated using a 2-factorial ANOVA [frequency x intensity] and inspection of the signal-to-noise ratio. Signal-to-noise ratio contrasted the neural response after sound onset (10ms – 150ms) with the average baseline activity 200ms before stimulus presentation. A 50ms sliding window was used to estimate SNR across time. Signal-to-noise onset ratio of the recording site was then defined as the average difference between activity across all sliding window positions and baseline measurements. Recording sites were included if the signal-to-noise ratio across all trials exceeded 3 and if the ANOVA yielded a frequency specific effect (p < 0.05). Trials with movement artifacts, identified by saturated LFP signal, were excluded from further analysis.

The best frequency of a site was determined by taking the maximum of the average response to pure tones across trials for each condition [frequency x intensity]. The data were then averaged across sound intensities, smoothed with a smoothing spline (smoothing parameter 0.98) and the best frequency was assigned to the peak of the resulting curve. Spatial maps were created by rounding recording coordinates to integers and averaging best-frequencies and peak latencies of all included recording sites for each coordinate. Resulting maps were smoothed with a 2x2mm Gaussian kernel. Coordinates beyond the edges of the recording field were not taken into account for smoothing. The tonotopic low frequency gradient reversal was used to subdivide the recording field into the anterior and posterior area. The boundary was placed between the two pixels with the lowest average best frequency in anteroposterior direction.

In order to identify phase-locking capabilities of recording sites, LFP responses to 200ms long click trains of 4 different frequencies (25, 50, 75, 100Hz) were analyzed. A fast Fourier transform (FFT) was performed on both baseline- and stimulus presentation period. Stimulus-evoked spectral power was then compared to a threshold that was defined as the mean spectral power during the baseline period plus two standard deviations. If the stimulus-evoked LFP power exceeded this threshold at the click train frequency, responses of that channel were labeled ‘phase-locked’. The strength of the phase-locking capability was further quantified by comparing to how many different click trains the LFP response phase-locked to.

The assessment of areal membership of recorded sites was based on structural MRI, tonotopy, peak latencies to pure tones and either histology (M1) or responses to click trains (M2).

For the figure-detection task, a minimum of 10 repetitions per stimulus was required for inclusion. Recording sites that were sound responsive and showed a significant difference between average MUA in hit and correct rejection trials (2-sample t test, p < 0.01) were classified as modulated and included into the analysis. Specifically, the 2-sample t test compared the average MUA in response-aligned figure trials (300-100ms window before the behavioral response) with a pseudo-randomly assigned, 200ms long time window (based on the figure onset distribution) in control trials. Sound responsiveness was assessed by comparing the spectral power during sound presentation and baseline period. Only if the neuronal response showed a power increase at the chord presentation rate (50ms duration of SFG stimulus chord → 20Hz) that was at least two times the baseline power of that frequency, sites were classified as sound responsive.

To determine the onset of the figure-ground modulation, the mean MUA for each SFG stimulus time bin (1ms bin width) was extracted. Subsequently, a difference curve for a given figure stimulus was calculated with all control stimuli that were presented (Fig_x_ – Ctr_1:n_). The resulting 96 difference curves (12 figure stimuli ^∗^ 8 Control stimuli) were then pooled and the mean MUA difference for each time bin was bootstrapped (5000 repetitions). We defined the onset of the figure-ground effect as the first significant sample (p < 0.01) after figure onset that was followed by at least four consecutively significant time bins (5ms in total).

To quantify how reliable recording sites can discriminate between figure and control trials we calculated neuronal d-prime: d_AB_ = (m_A_–m_B_)/s, where m_A_ and m_B_ are the baseline-normalized, mean responses in stimulus conditions A and B, and s is the pooled standard deviation. This measure is equivalent to the standardized mean difference that we reported for each statistical test. In addition to this parametric measure, we also illustrate the non-parametric area under the receiver operating characteristics (AUROC) that demonstrate figure-ground modulation based on a binary classifier.

Statistical tests between different stimulus conditions were done using a paired, two-sided Wilcoxon signed rank test. P values were false-discovery-rate (FDR) corrected. Please note that statistical details can be found in the figure legends.

## Supplemental information

Document S1. Figures S1–S5

Document S2. Article plus supplemental information
