# Supplementary material for: Neuronal figure-ground responses in primate primary auditory cortex
Source: Cell Rep. 2021 Jun 15;35(11):109242. doi: 10.1016/j.celrep.2021.109242 (PMC8220257; doi:10.1016/j.celrep.2021.109242)
Supplement: Document S1. Figures S1–S5 [file mmc1.pdf]

Cell Reports, Volume *35*

Supplemental information

Neuronal figure-ground responses

in primate primary auditory cortex

Felix Schneider, Fabien Balezeau, Claudia Distler, Yukiko Kikuchi, Jochem van Kempen, Alwin Gieselmann, Christopher I. Petkov, Alexander Thiele, and Timothy D. Griffiths

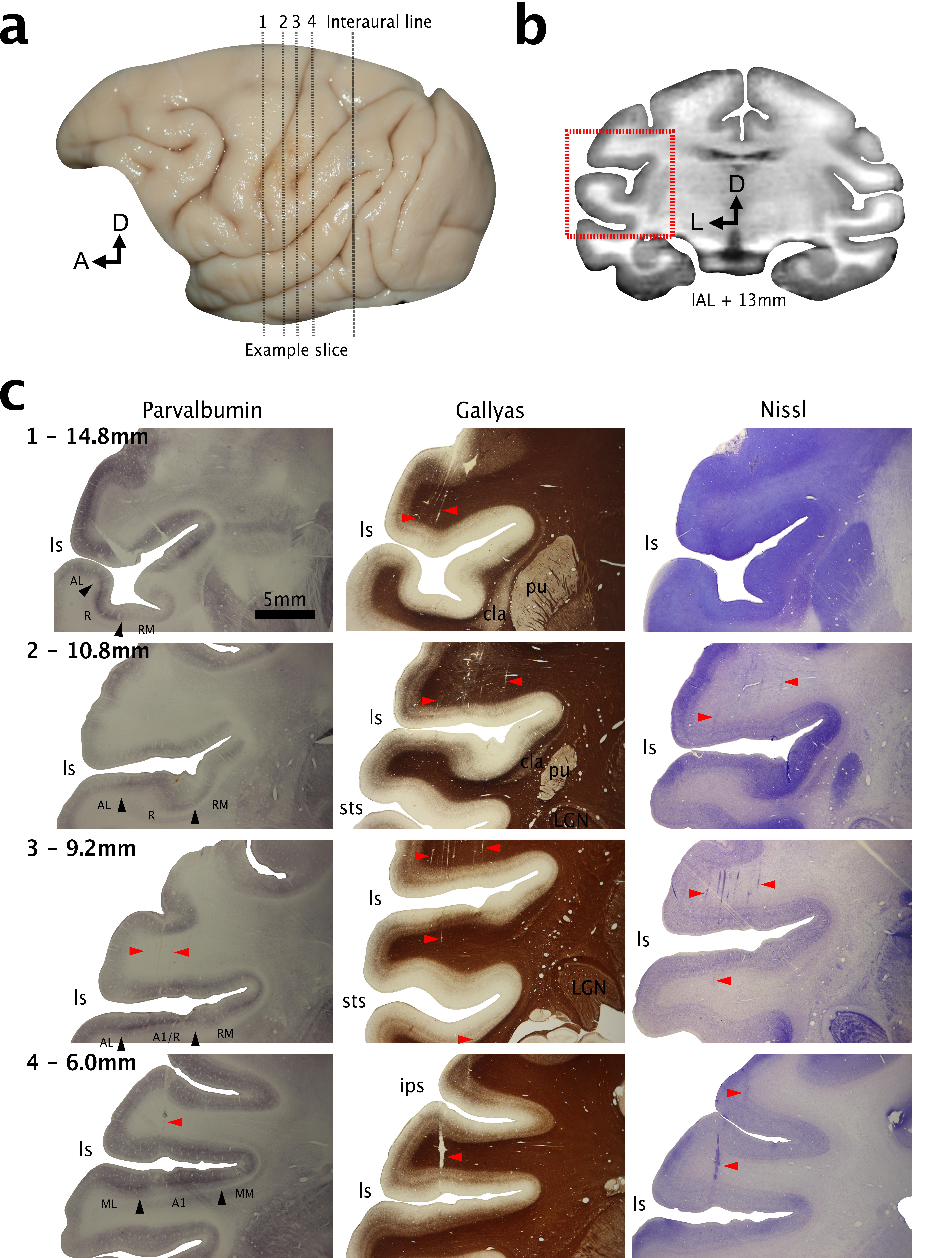
a
1 2 3 4 Interaural line
D
A
Example slice
b
D
L
IAL + 13mm
c
Parvalbumin
Gallyas
Nissl
1 - 14.8mm
ls
AL
R
RM
5mm
pu
cla
2 - 10.8mm
AL
R
RM
cla
pu
sts
LGN
3 - 9.2mm
AL
A1/R
RM
LGN
4 - 6.0mm
ML
A1
MM
ips

Supplementary Figure 1: Histology of M1. Related to Figure 1.

(**a**) Extracted brain with marked interaural line (dashed) and approximated slice location (dotted). A macaque brain atlas (Saleem & Logothetis, 2012) was used to align the example slice locations with the brain. Anatomical markers such as the shape of the superior temporal plane, IPS and the Claustrum served as a guide. (**b**) Coronal MR image (T1), approximately 13mm anterior to the interaural line. Red box indicates field of view for brain slices below. (**c**) Stained, coronal brain slices (50um each) showing parts of the left parietal and temporal lobe. Approximated distance from interaural line is indicated in black. Note that these distances were measured after the brain has shrunk due to the preservation procedure. Electrode traces marked with red arrows. Approximated core boundaries are indicated by black arrows. Abbreviation: cla, Claustrum; IAL: Interaural line; ips, Intraparietal Sulcus; LGN, Lateral geniculate nucleus; ls, Lateral Sulcus; pu, Putamen; Auditory areas: A1, Primary auditory cortex; AL, anterolateral belt; ML, Middle lateral belt; MM, Middle medial belt; R, Rostral core; RM, Rostromedial belt

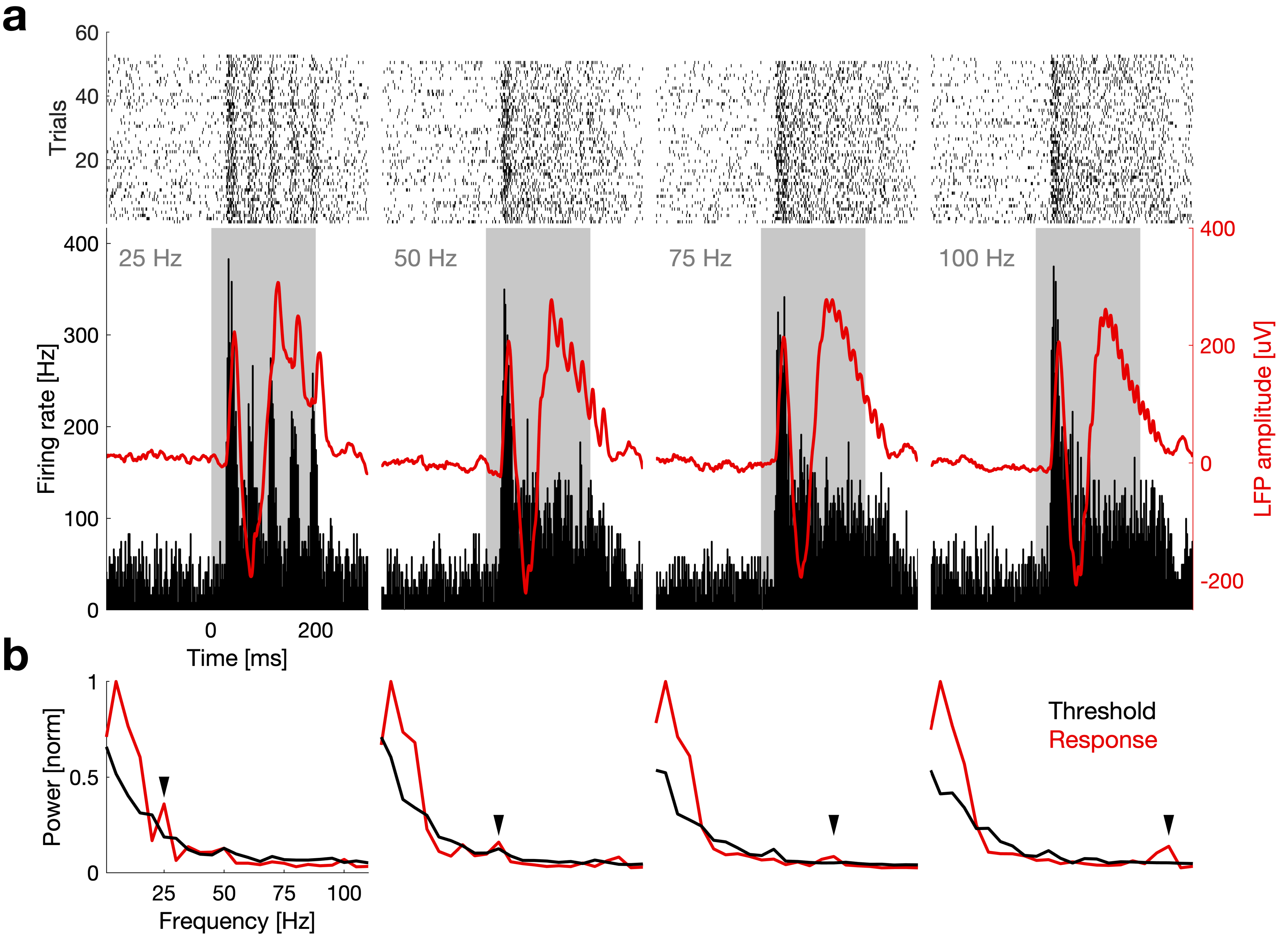
a
Trials
60
40
20
25 Hz
50 Hz
75 Hz
100 Hz
Firing rate [Hz]
400
300
200
100
0
LFP amplitude [uV]
400
200
0
-200
0
200
Time [ms]
b
Power [norm]
1
0.5
0
25
50
75
100
Frequency [Hz]
Threshold
Response

Supplementary Figure 2: Spiking and local field potential (LFP) response of an example site to click trains. Related to Figure 1.

(**a**) Raster plots are shown on top. Each row corresponds to a trial, each point within a trial to a single spike. Different plots correspond to different stimulus frequencies. 60 repetitions were presented per click train frequency. Trials with motion artefacts were excluded. PSTH with a 2ms bin width plotted below. Overlaid red line shows raw LFP trace. Shaded areas indicate the stimulus presentation period. Click train frequency is indicated in grey. (**b**) Average FFT of raw LFP response to click trains during stimulus presentation period. Click train frequency is indicated by the black arrow. Significance threshold (black line) was determined by calculating the spectral power of the baseline period and adding two standard deviations. For this example site, all click rates (4/4,100%) induced phase-locked responses.

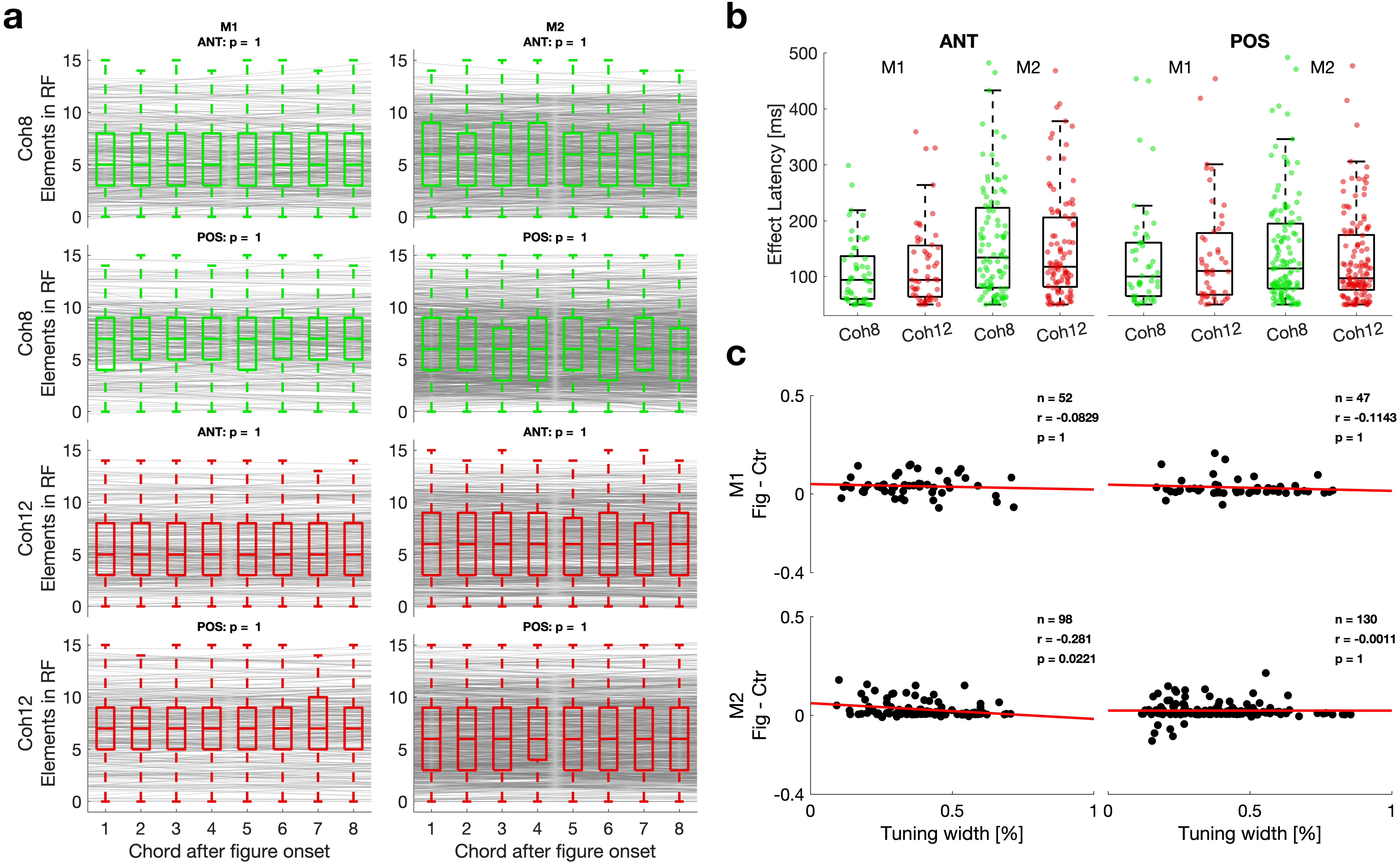

a
M1
ANT: p = 1
M2
ANT: p = 1
POS: p = 1
POS: p = 1
ANT: p = 1
ANT: p = 1
POS: p = 1
POS: p = 1
Coh8
Elements in RF
Coh12
Elements in RF
Chord after figure onset
b
ANT
POS
M1
M2
M1
M2
Effect Latency [ms]
Coh8
Coh12
Coh8
Coh12
Coh8
Coh12
Coh8
Coh12
c
n = 52
r = -0.0829
p = 1
n = 47
r = -0.1143
p = 1
n = 98
r = -0.281
p = 0.0221
n = 130
r = -0.0011
p = 1
M1
Fig - Ctr
M2
Fig - Ctr
Tuning width [%]
Tuning width [%]

Supplementary Figure 3: Summary of frequency content in receptive field, modulation latencies and tuning width. Related to Figure 2 and Figure 3.

(**a**) Quantification of frequency elements that fall into the frequency-selective area (receptive field, RF) of each recording site for the first eight chords after figure onset. Data are shown for each coherence level (Coh8: green, Coh12: red), recording field (anterior vs posterior) and subject. The half-maximum was used to classify pure tone responses into either the frequency-selective or unresponsive category. For each individual stimulus, the number of frequency elements that fall into the RF of the recording site was counted for the first eight chords after figure onset, which was the basis for a linear regression (shown in grey here). The slopes of this regression were tested against zero (Wilcoxon signed rank test). The resulting FDR-corrected p-values are displayed above. (**b**) Figure-ground effect latency for anterior (left) and posterior recording region (right) for both subjects. Modulation latencies for figures with 8 (green) and 12 coherent elements (red) shown for recording sites with significant figure vs control response ($p < 0.01$). Latencies were determined by bootstrapping the mean MUA of each time bin (5000 repetitions). The modulation onset was defined as the first significantly different time bin that was followed by at least four additional significantly different time bins (5ms, $p < 0.01$). The smallest possible latency value was set to 50ms (Duration of one chord). Data points are shown for sessions where this procedure was able to extract a latency value. (**c**) Correlation between tuning width and figure-ground modulation for M1 (top) and M2 (below). Anterior (left) and posterior field (right) shown. MUA differences between figure and control condition plotted as a function of tuning width, expressed as percentage of the tested frequency space. Each point represents one recording site. Data are pooled across coherence conditions. The red line shows the least-squares line. Correlation coefficient and p-value are shown within each plot.

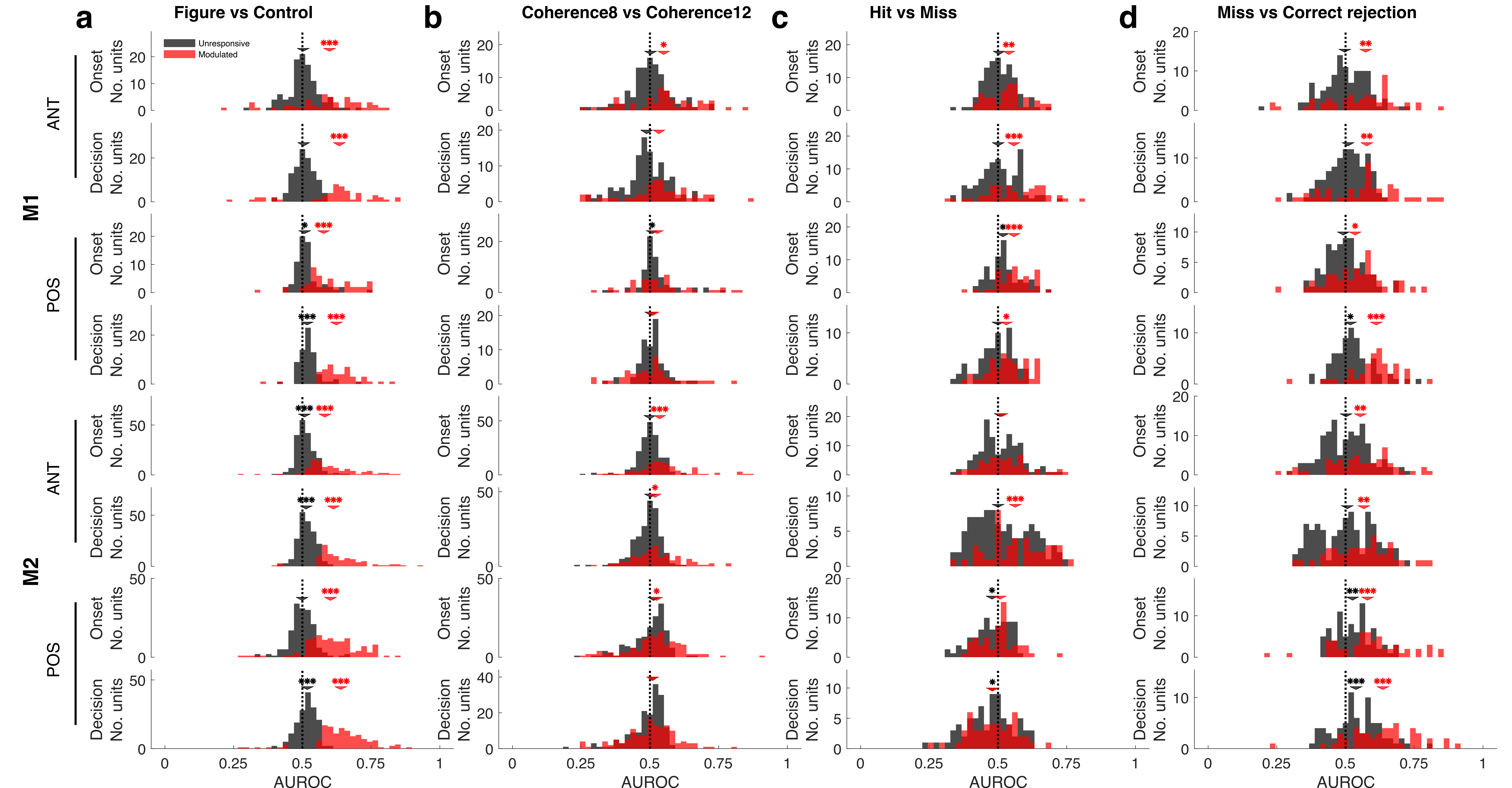

a
Figure vs Control
b
Coherence8 vs Coherence12
c
Hit vs Miss
d
Miss vs Correct rejection
Unresponsive
Modulated
M1
M2
ANT
POS
Onset
Decision
No. units
AUROC

Supplementary Figure 4: Subject-wise analysis of neuronal modulation. Related to Figure 4.

Area under receiver operating characteristic (AUROC) distributions shown for both anterior and posterior recording field of M1 (top) and M2 (below). Significantly figure-modulated MUA is shown in red. Remaining unresponsive sites are shown in black. (**a**) Figure-ground modulation. (**b**) Modulation based on figure coherence. (**c**) Modulation based on behavioural detection of temporally coherent elements. Only recordings with at least 20 miss trials were included (response defined as end of figure). (**d**) Modulation based on temporal coherence without detection. Arrows indicate median of distribution. Data were tested against 0.5 with a two-sided Wilcoxon signed rank test. All p-values are FDR-corrected. Colour-coded stars indicate significance: * $p < 0.05$, ** $p < 0.01$, *** $p < 0.001$.

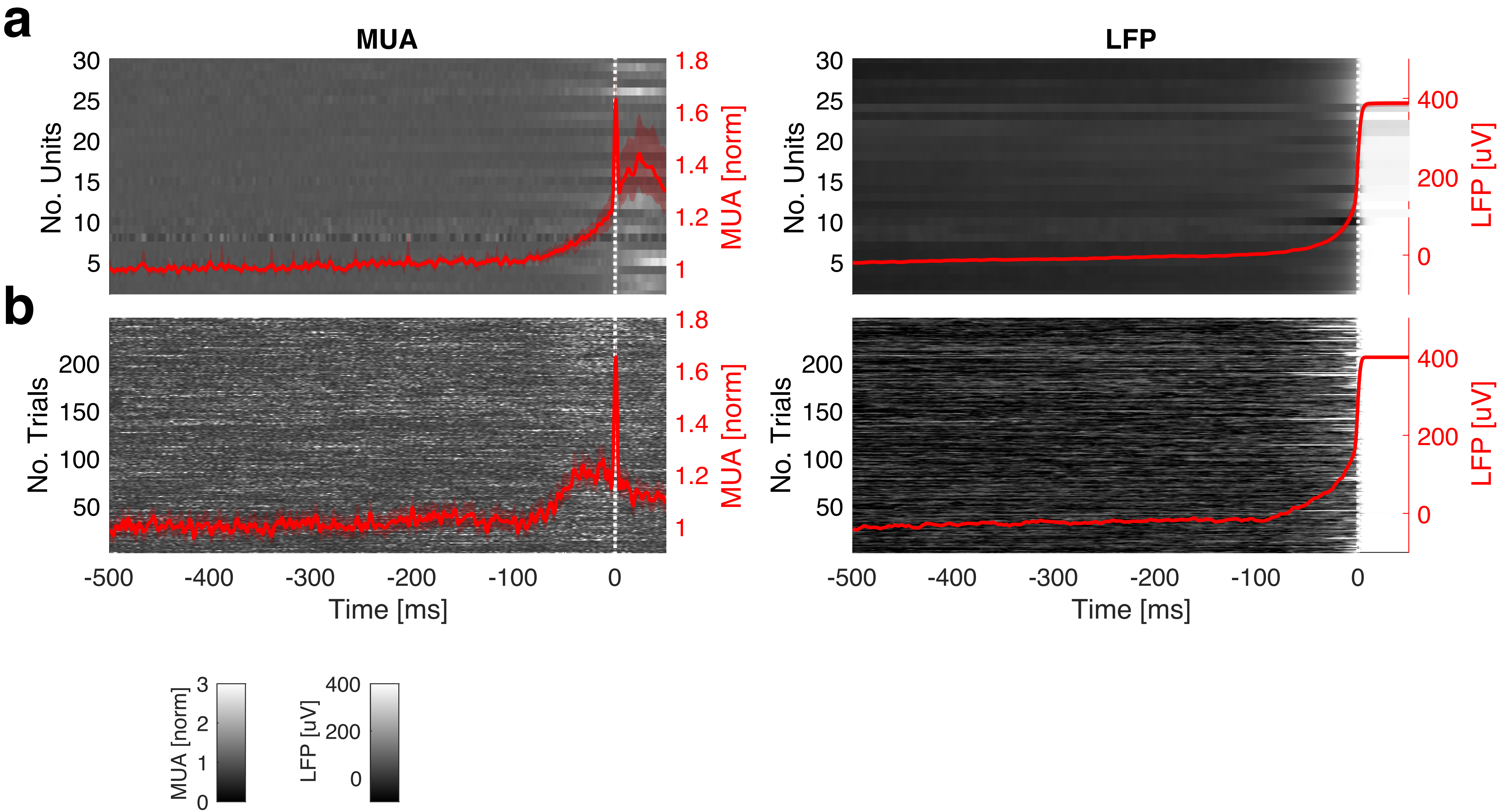
a
MUA
LFP
b
No. Units
No. Trials
MUA [norm]
LFP [uV]
Time [ms]
-500
-400
-300
-200
-100
0
1.8
1.6
1.4
1.2
1
400
200
0
30
25
20
15
10
5
200
150
100
50
3
2
1

Supplementary Figure 5: Self-paced touch bar release experiment. Related to Figure 4.

(**a**) Movement-aligned multi-unit activity (left) and LFP responses (right) of 30 sound-driven recording sites. The colour coding shows the average activity of each channel. The red line corresponds to the average activity across channels. The shaded area illustrates 95% confidence intervals. The white dashed line marks the touch bar release. (**b**) Multi-unit and LFP activity of an example channel. Same conventions as in (**a**).
